# Response of swine spleen to *Streptococcus suis *infection revealed by transcription analysis

**DOI:** 10.1186/1471-2164-11-556

**Published:** 2010-10-11

**Authors:** Ran Li, Anding Zhang, Bo Chen, Liu Teng, Ya Wang, Huanchun Chen, Meilin Jin

**Affiliations:** 1 Unit of Animal Infectious Diseases, National Key Laboratory of Agricultural Microbiology, Huazhong Agricultural University, Wuhan, Hubei, China; 2 College of Veterinary Medicine, Huazhong Agricultural University, Wuhan, Hubei, China

## Abstract

**Astract:**

**Background**

*Streptococcus suis *serotype 2 (SS2), a major swine pathogen and an emerging zoonotic agent, has greatly challenged global public health. Systematical information about host immune response to the infection is important for understanding the molecular mechanism of diseases.

**Results**

104 and 129 unique genes were significantly up-regulated and down-regulated in the spleens of pigs infected with SS2 (WT). The up-regulated genes were principally related to immune response, such as genes involved in inflammatory response; acute-phase/immune response; cell adhesion and response to stress. The down-regulated genes were mainly involved in transcription, transport, material and energy metabolism which were representative of the reduced vital activity of SS2-influenced cells. Only a few genes showed significantly differential expression when comparing avirulent isogenic strain (ΔHP0197) with mock-infected samples.

**Conclusions**

Our findings indicated that highly pathogenic SS2 could persistently induce cytokines mainly by Toll-like receptor 2 (TLR2) pathway, and the phagocytosis-resistant bacteria could induce high level of cytokines and secrete toxins to destroy deep tissues, and cause meningitis, septicaemia, pneumonia, endocarditis, and arthritis.

## Background

*Streptococcus suis *(*S. suis*) is an important pathogen associated with many diseases in pigs, including meningitis, septicaemia, pneumonia, endocarditis, and arthritis. *S. suis *serotype 2 (SS2) is considered the most pathogenic as well as the most prevalent capsular type among thirty-three serotypes (types 1 to 31, 33, and 1/2) in diseased pigs, and it is also the causative agent of serious infections in humans, especially in people in close contact with pig or pork byproducts [1-3]. Two recent large-scale outbreaks of human SS2 epidemics in China (one had 25 cases with 14 deaths in Jiangsu in 1998, the second had 204 cases with 38 deaths in Sichuan in 2005), featured clinical streptococcal toxic shock syndrome, have greatly challenged the global public health [4-7]. Recently, *S. suis *infection has also caused sporadic human illness in other countries, including Thailand [8,9], United Kingdom [10], Portugal [11], Australia [12], Netherlands [13] and United States [14,15], and been recognized as the third most common cause of community acquired bacterial meningitis in Hong Kong and as the leading cause of adult meningitis in Vietnam [5,16].

The past pathogenesis studies were performed mainly on the pathogenic bacteria and as a result, a few virulence-associated factors have been successfully identified. Polysaccharide capsule has been considered essential for the virulence of the bacterium [17,18], and other factors, such as suilysin, the so-called extracellular protein factor and muramidase-released protein have been shown to be linked to, but not essential for the full virulence of *S. suis *[19]. GapdH[20], Enolase[21,22], FbpS[19], Adhesin [23-27] have been proved to be involved in the adherence and virulence of *S. suis*. Recently, serum opacity-like factor [28], IgA1 protease[29], D-Alanylation of Lipoteichoic Acid [30] and pgdA [31] were identified as important factors in *S. suis *virulence. In addition, SalK/SalR [32] and CovR [33] were found to affect the virulence of *S. suis *Chinese isolates. These studies have contributed to the understanding of *S. suis *pathogenesis and also suggested that host responses also play essential roles in the development of the diseases.

Inducing excessive inflammation is recognized as one of the reasons why highly invasive SS2 strain could cause severe diseases [31,34]. A few previous studies indicated that high level of cytokines and chemokines could be released by human brain microvascular endothelial cells [35], a whole-blood culture system [36], macrophages [37] and monocytes [38] stimulated by SS2, and have important roles in the initiation and development of inflammation and meningitis [39]. More direct proofs were the studies on mice with different genetic background, which indicated that IL-10 was responsible, at least in part, for the high survival, which suggested that aberrant innate immune response contributed to SS2 diseases [40].

To be aware of the information about host immune response would enable people to better understand the disease. Transcriptional response of alveolar macrophages to SS2 has been performed and the results indicated that NF-kB and MAP-kinases signaling pathways were induced upon interaction with SS2 [41]. However, it is not easy to get more information since the primary macrophages are so sensitive to the interference. Spleen plays an important role in immune response and could be an ideal target to study host immune response against infection [42,43]. In the present study, the gene expression profiles of swine spleens which suffered from highly pathogenic SS2, avirulent isogenic strain and PBS respectively were investigated to reveal the host immune response to SS2 and the contributions of host response to SS2 diseases.

## Results

### Transcriptome analysis

**Figure 1:**
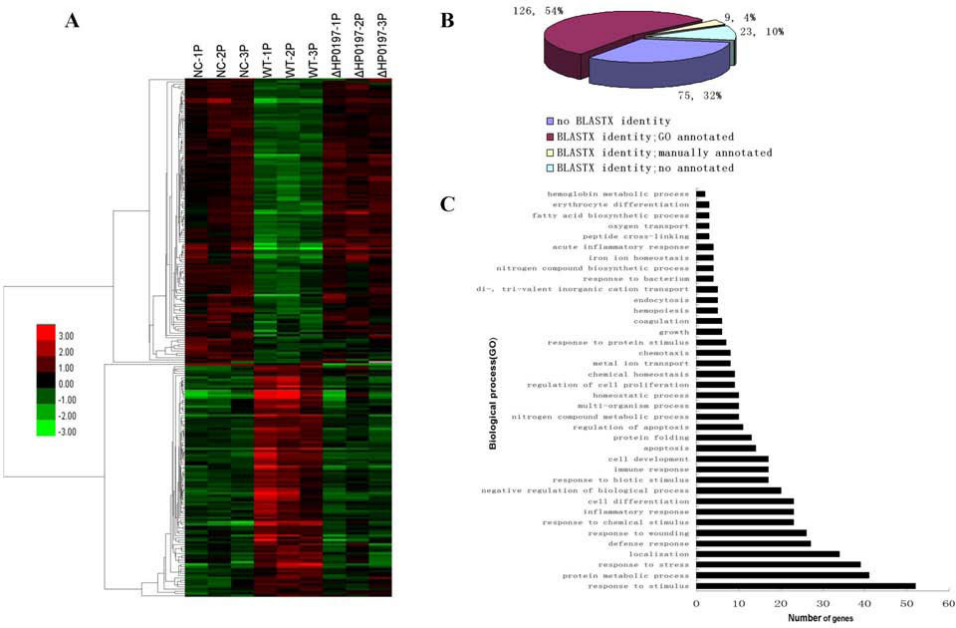
**Clustering and characterization of the differential expression of genes**. (A) 233 genes were selected for cluster analysis which is described in methods. Each row represents a separate transcript and each column represents a separate piglet. Color legend is on the left, the color scale ranges from saturated green for log ratios -3.0 and above to saturate red for log ratios 3.0 and above. Red indicates increased transcript expression levels, green indicates decreased levels compared with normal samples. (B) Percentage distribution of unique genes was from 233 differentially regulated transcripts after BLASTX searches and annotation. 158 unique genes had significant similarities based on BLASTX searches. 135(126+9) unique genes had been annotated by Biological Process (BP) Classification. (C) Categories of annotated genes genes based on biological process GO term. Many categories shared the same transcripts.

The transcriptome analysis indicated that 14,992, 15,487 and 15,757 probe sets, corresponding to 62.1%, 64.2% and 65.3% of all probe sets, were detected in WT, ΔHP0197 and mock-infected pig spleens respectively (Additional file 1). The expression profiles of porcine spleens challenged with WT 3 days post inoculation were compared with those of the mock-infected group. After quantile normalization and statistical analysis, 1014 transcripts were identified at the global false discovery rate (FDR) of 10% (Additional file 2). Furthermore, the criteria of a two-fold or greater change in differential expression and a FDR of 10% were chosen to determine up-regulated and down-regulated genes in the WT infected replicates. Using these criteria, 120 and 132 transcripts, representing 104 and 129 unique genes, were significantly up-regulated and down-regulated respectively (Additional file 3). However, only a few genes showed significantly differential expressions when comparing ΔHP0197 with mock-infected samples (Figure 1A).

Of the 233 unique DE transcripts, 158 transcripts could be determined based on BLASTX searches and annotated with DAVID or by searching against the GenBank database (Figure 1B). Among these, 135 unique genes were grouped into 39 categories based on biological process Gene Ontology (GO) terms or according to their potential Biology Process Classification by referring to recent publications (Figure 1C). Unsurprisingly, the majority of genes were related to the immune response, Transcription, Transport, material and energy metabolism, etc. (Table 1).

**Table 1:** Different expression of genes in spleens after *S. suis *infection 3 days

Function classification	ENTREZ GENE_ID	Description	Fold change	Q-value (%)
**Inflammatory response**				
	929	CD14 Antigen	3.4	1.222
	6279	S100 Calcium binding protein A8	19.3	1.508
	6280	S100 Calcium binding protein A9	16.1	0
	3588	Interleukin 10 receptor, beta	2.7	3.911
	5743	Prostaglandin-Endoperoxide synthase 2	4.7	7.146
	7057	Thrombospondin 1	2.4	6.387
	3576	Interleukin 8	5.6	6.387
	9547	Chemokine (C-X-C Motif) Ligand 14	2.0	8.898
	6283	S100 Calcium binding protein A12	18.6	0
	2908	Nuclear receptor subfamily 3, group C, member 1	0.5	6.882
	6363	Chemokine (C-C Motif) Ligand 19	2.2	1.508
	7097	Toll-like receptor 2	2.0	6.387
	2920	Chemokine (C-X-C Motif) Ligand 2	7.3	3.385
	246	Arachidonate 15-Lipoxygenase	0.2	2.181
	7052	Transglutaminase 2	2.1	6.387
	9332	CD163 antigen	11.7	0
	6288	Serum amyloid A1	6.4	1.222
	3553	Interleukin 1, beta	16.7	6.387
	3569	Interleukin 6 (Interferon, Beta 2)	4.8	6.387
	56729	*Resistin*	3.7	7.146
**Response to stress**				
	1153	Cold inducible rna binding protein	0.43	5.612
	3320	Heat shock protein 90Kda alpha class A member 1	3.2	8.898
	6916	Thromboxane A synthase 1	2.6	3.911
	10963	Stress-induced-phosphoprotein 1	2.5	0
	130872	AHA1, activator of heat shock 90Kda protein ATPase homolog 2	2.6	1.508
	3337	Dnaj (Hsp40) homolog, subfamily B, member 1	2.8	1.222
	871	Serpin peptidase inhibitor, clade H (Heat Shock Protein 47), member 1	2.7	1.222
	10808	Heat shock 105Kda/110Kda protein 1	3.3	0
	3301	Dnaj (Hsp40) homolog, subfamily A, member 1	2.4	0
	3304	Heat Shock 70Kda Protein 1A	11.1	0
**Coagulation**				
	5328	Plasminogen activator, urokinase	2.0	6.387
	2162	Coagulationfactor XIII, A1 polypeptide	6.8	3.385
**Signal transduction**				
	9465	A kinase anchor protein 7	0.5	6.698
	8519	Interferon induced transmembrane protein 1	2.0	6.387
	115265	Dna-damage-inducible transcript 4-like	0.3	2.181
	9770	Ras association (Ralgds/Af-6) domain family 2	2.4	6.387
	9510	Adamm etallopeptidase with thrombospondin type 1 motif, 1	2.3	0
	1363	Carboxypeptidase E	0.44	6.698
	54210	Triggering receptor expressed on myeloid cells 1	3.2	6.387
	9289	G protein-coupled receptor 56	0.46	6.698
	7043	Transforming growth factor, beta 3	2.1	4.116
**Transcription**				
	2353	V-Fos fbj murine osteosarcoma viral oncogene homolog	2.8	3.911
	84969	Chromosome 20 open reading frame 100	0.3	2.541
	55885	Lim domain only 3	0.3	2.181
	3726	Jun B proto-oncogene	2.6	7.146
	91	Activin a receptor, type ib	3.4	1.222
	116448	Oligodendrocyte transcription factor 1	2.4	6.387
	79365	Basic helix-loop-helix domain containing, class B, 3	0.4	2.181
	64919	B-cell cll/lymphoma 11B	0.5	2.181
	23635	Single-stranded dna binding protein 2	0.4	2.541
	23414	Zinc finger protein, multitype 2	0.5	6.698
	7552	Zinc finger protein 6 (Cmpx1)	0.5	3.385
	6920	Transcription elongation factor A (SII), 3	2.2	6.387
	4783	Nuclear factor, interleukin 3 regulated	2.4	3.911
	1052	CCAAT/Enhancer binding protein (C/EBP), delta	3.1	0
**Cell adhesion**				
	6401	Selectin E	3.5	1.222
	8174	Mucosal vascular addressin cell adhesion molecule 1	2.3	6.387
	5067	Contactin 3	0.5	6.794
	4867	Nephronophthisis 1 (Juvenile)	0.4	3.911
	1462	Chondroitin sulfate proteoglycan 2 (Versican)	9.1	0
	960	CD44 antigen	2.3	2.541
**Ubiquitin cycle**				
	115123	Membrane-associated ring finger (C3HC4) 3	4.4	1.222
	7317	Ubiquitin-activating enzyme E1	0.4	2.181
	11274	Ubiquitin specific peptidase 18	0.4	6.698
	9666	Zinc finger daz interacting protein 3	0.5	6.882
**Transport**				
	6556	Solute carrier family 11, member 1	4.0	2.051
	4057	Lactotransferrin	5.9	3.385
	1356	Ceruloplasmin (Ferroxidase)	2.3	2.541
	1410	Crystallin, alpha B	2.8	6.387
	283652	Solute carrier family 24, member 5	0.4	2.181
	54843	Synaptotagmin-like 2	0.4	6.698
	6947	Haptocorrin	8.9	0
	3949	Low density lipoprotein receptor	2.1	5.612
	3043	Hemoglobin, beta	0.4	6.698
	3042	Hemoglobin, alpha pseudogene 2	0.2	2.181
	3040	Hemoglobin, alpha 1	0.2	2.181
	2554	Gamma-aminobutyric acid (Gaba) a receptor, alpha 1	0.3	3.911
	2288	Fk506 binding protein 4, 59Kda	2.2	1.222
	6557	Solute carrier family 12, member 1	0.4	6.882
	152789	*Janus kinase and microtubule interacting protein 1*	0.5	6.794
**Nucleic acid metabolic process**				
	401251	Muts homolog 5	0.5	6.698
	56952	Phosphoribosyl transferase domain	0.4	2.181
	51251	5'-Nucleotidase, cytosolic Iii	0.4	6.698
	10492	Synaptotagmin binding, cytoplasmic rna interacting protein	0.4	4.116
	8347	Histone 1,H2bd	2.6	6.387
	8334	Histone 1, H2ac	5.6	1.222
	6430	Splicing factor, arginine/serine-rich 5	0.5	0
	4302	Myeloid/Lymphoid or mixed-lineage leukemia translocated to, 6	0.4	6.882
**Response to stimulus**				
	5806	Pentraxin-related gene, rapidly induced by il-1 beta	14.1	1.222
	6372	Chemokine (C-X-C motif) ligand 6	5.5	1.222
	64135	Interferon induced with helicase c domain 1	0.4	6.698
	3240	Haptoglobin	4.6	0
	6648	Superoxide dismutase 2, mitochondrial	4.6	1.508
	1843	Dual specificity phosphatase 1	2.1	6.387
**Cell differentiation/development**				
	58189	Wap four-disulfide core domain 1	2.1	1.222
	9531	Bcl2-associated athanogene 3	3.7	0
	51454	Gulp, engulfment adaptor ptb domain containing1	0.4	2.181
	212	Aminolevulinate, delta-, synthase 2	0.4	6.698
	2012	Epithelial membrane protein 1	2.0	7.146
	79689	Steap family member 4	2.8	3.911
	9021	Suppressor of cytokine signaling 3	2.4	3.385
	5270	Serpin peptidase inhibitor, clade E, member 2	2.3	3.385
	1946	Ephrin-A5	0.4	3.911
	85444	*Leucine rich repeat and coiled-coil domain containing 1*	0.5	5.612
	54873	Palmdelphin	0.4	5.612
	10439	Olfactomedin 1	3.3	6.387
**Carbohydrate metabolic process**				
	4199	Malic enzyme 1, NADP(+)-dependent, cytosolic	0.4	3.911
	80760	Inter-alpha (Globulin) inhibitor H5	0.3	6.698
	152831	Klotho beta	2.5	0
	3101	Hexokinase 3 (White Cell)	2.9	6.387
	3099	Hexokinase 2	2.3	8.898
	1116	Chitinase 3-like 1	2.9	8.898
**Protein metabolic process**				
	85464	Slingshot homolog 2	2.3	1.508
	51327	Erythroid associated factor	0.06	0
	7076	Timp metallopeptidase inhibitor 1	4.4	1.222
	7053	Transglutaminase 3	6.1	1.222
	114907	F-box protein 32	0.4	6.794
	64844	Membrane-associated ring finger (C3HC4) 7	0.5	2.181
	64172	O-sialoglycoprotein endopeptidase-like 1	0.4	2.181
	55466	Dnaj (Hsp40) homolog, subfamily A, member 4	2.7	2.541
	51056	Leucine aminopeptidase 3	2.0	8.898
	2289	Fk506 binding protein 5	2.5	6.387
	26235	F-box and leucine-rich repeat protein 4	0.5	6.698
**Nitrogen compound metabolic process**				
	383	Arginase	0.2	2.181
	64850	Alanine-glyoxylate aminotransferase 2-like 1	0.2	6.882
	6799	Sulfotransferase family, cytosolic, 1A, phenol-preferring, member 2	2.0	6.387
	8974	Procollagen-proline, 2-oxoglutarate 4-dioxygenase, alpha polypeptide ii	3.2	6.387
**Others**				
	129446	*Cardiomyopathy associated 3*	0.3	2.181
	128218	*Transmembrane protein 125*	2.0	8.898
	57763	*Ankyrin repeat, family A, 2*	0.4	2.181
	29970	*Schwannomin interacting protein 1*	0.5	6.794
	23336	*Desmuslin*	0.5	6.882
	590	Butyrylcholinesterase	0.5	4.116
	84649	Diacylglycerol o-acyltransferase homolog 2	3.2	1.222
	79887	*Hypothetical protein Flj22662*	3.5	6.387

DE genes which putative functions assigned based on GO term and manual annotation. Manual annotations were listed in italics. Many genes with multiple functions were only listed in one category according to specific biology processes. "FC≥2" represents up regulation (infection/control), "FC ≤ 0.5" represents down regulation.

### Validation of microarray data by quantitative real-time PCR (qPCR)

The qPCR was performed to validate the expression patterns during infection for specific genes identified in the microarray assay. In order to validate the differential expression of various identified genes, 16 up-regulated genes, with the increase ranging from 2.0-fold to 18.6-fold, and 3 down-regulated genes, with the decrease ranging from 2.5-fold to 5.9-fold, were selected for qPCR analysis. All the selected down-regulated genes could be amplified from the control samples but failed to achieve significant detectable signs from WT-infected spleens, except for *ALOX15 *which showed 3.2-fold down-regulated expression. All selected up-regulated genes showed higher expression in WT-infected samples than in the control samples (Table 2). Though variation in fold changes could be observed between qPCR and microarray (Table 2), the differential expression patterns were coincident between the results of the two techniques, which indicated the reliability of the microarray analysis.

**Table 2:** Validation of microarray results by qPCR

Gene	Accession	Microarray fold change	qPCR fold change (p-value)
*IL1B*	CK468468	16.7	258.3 (0.0117)
*S100A9*	BI402402	16.1	137.2(0.0007)
*S100A12*	CB475695	18.6	76.7 (< 0.0001)
*HSP90*	CF180819	3.18	48.5 (0.0321)
*IL8*	NM_213867	5.58	35.5 (0.0066)
*HSP70*	NM_213766	11.06	31.4 (0.0099)
*TIMP1*	NM_213857	4.4	14.6 (0.0023)
*IL6*	AF493992	4.8	10.5 (0.0074)
*SOD2*	NM_214127	4.6	10.0 (< 0.0001)
*NRAMP1*	U55068	3.97	7.2 (0.0035)
*SELE*	NM_214268	3.5	5.9 (0.0002)
*PLAU*	NM_213945	2.0	5.5 (0.0415)
*CCL19*	BX672579	2.16	4.2 (0.0004)
*haptocorrin*	CB472702	3.78	2.1(0.0388)
*TLR-2*	NM_213761	2.0	2.1 (< 0.0001)
*ALOX15*	NM_213931	0.2	0.3 (0.038)

### Induction of inflammasomes and acute phase proteins by SS2 infection

Highly pathogenic SS2 infection could cause up-regulated expression of a large set of genes involved in the inflammatory response and acute phase proteins by microarray analysis. IL-1B, IL-6 and IL-8 could be induced by foreign pathogens and play essential roles in controlling infections [5,44]. However, they may also cause pathology when these productions are excessive or uncontrolled [45]. Ye et al. also found that significantly high level of cytokines could be induced by highly pathogenic SS2 strain and play important roles in sepsis [34], which is in coincidence with ours. In addition, quite a few genes related to inflammatory response were found up-regulated, such as S100 family proteins (S100A8, S100A9 and S100A12) [46], Pentraxin 3 [47] and Resistin [48,49]. They play important roles in mediating inflammatory responses, recruiting inflammatory cells to sites of tissue damage or contributing to resisting the invasion of various pathogens.

Acute phase proteins (APPs), such as Lactotransferrin [50], Haptoglobin [51], Serum amyloid A 2 [52] and coagulation factor XIII, were involved in physiologic reactions initiated early in the inflammatory process [53], and could be a response to S. sui*s *infection [54]. CEBPD belonging to the CCAAT-enhancer binding protein (CEBP) family which is crucial in the regulation of genes involved in immunity and inflammation. These up-regulated genes are the representative of host acute response struggling to eliminate invading pathogens.

### Induction of genes related in cell adhesion and stress response

Cell adhesion molecules (CAMs) have been implicated in the regulation of a wide variety of fundamental cellular processes, such as cell adhesion, cell polarization, survival, movement, and proliferation [55]. E-selectin is a cell adhesion molecule expressed on endothelial cells activated by cytokines, and plays an important role in recruiting leukocytes to the site of injury [56]. Versican can bind adhesion molecules on the surface of inflammatory leukocytes [57] and act as a TLR2 agonist in inducing the release of proinflammatory cytokines [58]. Thrombospondin 1 is an adhesive glycoprotein that mediates cell-to-cell and cell-to-matrix interactions and it could interact with numerous proteases involved in angiogenesis [59]. Mucosal vascular addressin cell adhesion molecule 1 is predominantly expressed on high endothelial venules in inflamed tissues, and could assist the extravasations of leucocyte [60]. The up-regulation of cell adhesion molecules after SS2 infection would contribute to recruiting leukocytes to the site of infection, which could control infection.

Genes related to oxidative stress and homeostasis were also identified to be up-regulated. SOD2 provides vital protection against reactive oxygen species (ROS), thus protecting tissues from damage in a broad range of disease states. The secretion of PGE2, together with nitric oxide production, is involved in disruption of the blood-brain barrier(BBB) in an experimental model of bacterial meningitis [61]. *S. suis*-mediated PGE2 production by human macrophages was also noticed by Jobin and contributed to the BBB disruption [62].

### Toll-like receptors (TLRs) pathway analysis

Activation of the innate immune response is controlled in large part by the Toll-like receptor (TLR) family of pattern-recognition receptors. The previous study showed that *S. suis *was mainly recognized via TLR2 by THP-1 monocytes, which was associated with CD14 [38] and led to the release of pro-inflammatory mediators [63]. The strong activation of TLR2 and CD14 was also observed in murine brain parenchyma after the presence of *S. suis *bacteremia [39]. A recent research indicated that components released during *S. suis *infection as well as penicillin-treated whole bacteria could induce NF-kB activation through TLR2/6 [64]. The obvious elevation of TLR2 (2.0 fold) and CD14 (3.4 fold) was noticed at transcript level in spleens after highly pathogenic SS2 infection. Unsurprisingly, MyD88, an adaptor molecule in downstream signaling events with TLRs and CD14, was up-regulated at the level of 1.5 fold (q < 10%). In contrast, the effect could not be seen with avirulent SS2 infection.

### Down-regulated transcripts following *S. suis *infection

The majority of down-regulated genes were related to transcription, transport, material and energy metabolism (Table 1). Highly pathogenic strain could show high level of toxicity to host cells [34], and as a result, the influenced cells could hardly to be active. So these down regulations could be regarded the representative of the reduced vital activity of SS2-influenced cells.

## Discussion

Two recent SS2 outbreaks in China not only seriously challenged public health but also shocked the scientific community, calling for the basic and translational studies of *S. suis*. Until now, several proteins were identified as vaccine candidates [65,66] and drug targets [67,68] for controlling SS2. In addition, emphasis is also extended to the pathogenesis study. Several pathogenic factors were successfully identified and strengthened the understanding for the virulence of the bacterium. As infectious disease resulted from the interplay between pathogens and the defense of the hosts they infect, host immune response was especially essential for understanding the diseases [41,69].

In the present study, we tried to compare the gene expression profiles of spleens from swine suffering from highly pathogenic SS2, from swine infected with the avirulent isogenic strain, and from swine inoculated with PBS respectively to reveal the host immune response to SS2 and the contributions of host response to SS2 diseases. It is not accidental that significant changes of gene expression profiles could be noticed when infected with highly pathogenic SS2 compared with mock-infected samples, while avirulent isogenic strain would cause similar profiles to mock-infected samples (Figure 1A). These indicated that avirulent isogenic strain could hardly cause significant gene expression which was coincident with the fact that no significant clinical symptoms could be noticed in pigs. Moreover, the obvious changes in gene expression profiles were highly associated with significant clinical signs on day 3 post-inoculation with highly pathogenic strain. Further analysis of the present study indicated that the majority of down-regulated genes were mainly involved in transcription, transport, material and energy metabolism which were representative of the reduced vital activity of SS2-influenced cells. However, the up-regulated genes were principally related to immune response, such as genes involved in inflammatory response; acute-phase/immune response; cell adhesion and response to stress. Undoubtedly, it would be meaningful to explore the roles of these genes in SS2-caused diseases.

First of all, it is necessary to know how SS2 induces immune response. It is well acknowledged that TLRs are transmembrane proteins that could recognize specific PAMPs and eventually result in the activation of NF-kB and MAP kinases to elicit regulatory response [70]. Among these transmembrane proteins, TLR-2 could recognize bacterial LAM, BLP and PGN by following their initial interaction with CD14. Previous reports indicated that *S. suis *mainly induced proinflammatory cytokines by TLR2 of human macrophages and murine brain [39,63], and several proinflammatory cytokines, such as IL-1B, IL-6, IL-8, TNF-a and MCP-1 could be triggered [35,36,38,41]. In our study, large doses of bacteria could be isolated from spleens of WT-infected pigs while no bacterium could be found to exist in pigs infected with ΔHP0197. In coincidence with these, TLR-2 pathway and several proinflammatory cytokines were induced only in WT-infected pigs. ΔHP0197 showed similar transcript profile as control pigs due to either failing to invading or being easily eliminated by host. In contrast, the large doses of bacteria effected maximal cytokines release in WT-infected pigs [37]. The exaggerated high levels of cytokines perhaps exacerbate the inflammation and were considered to be responsible for *S. suis *caused diseases [39]. So the successful lethal pathogens could persistently induce cytokines secreted originally to clear the foreign invader, and as a result, the host's defense was utilized by *S. suis *to cause diseases, and to some extent to death.

As we all know that the secreted cytokine is an important part of a host defense system, which could recruit inflammatory cells to sites of tissue damage and help to eliminate the pathogens. However, this innate defense system is a double edged sword. If the recruiting inflammatory cells could kill the invader, the disease could be controlled. On the opposite side, if the recruiting phagocytes could not efficiently kill the bacteria, the tide would be turned to pathogen's favor, and the persistently induced cytokines would result in the exacerbated inflammation and lead to the death during the septic phase of infection. These might be the reason why the survival rate could be elevated when inflammation was inhibited by IL-10 [40], and why the level of cytokine was correlated inversely with survival time in patients with sepsis [45]. In coincidence with our analysis, pathogenic *S. suis *could effectively resist the uptake by phagocytes and CPS could inhibit activation of signaling pathways involved in phagocytosis [17,71,72]. In addition, several virulence-associated proteins such as FBPS[19], PDGA[31], LTA[30], HP0197 (unpublished data), serine protease [73] etc. were also contributed to the phagocytosis resistance, and the up-regulation of these proteins in vivo may suggest the better phagocytosis resistance [31,74,75]. Due to failing phagocytosis, bacteria could not only cause exacerbated inflammation but also contribute to its survival in the bloodstream in "modified Trojan Horse" theory in which bacteria travel extracellularly while attached to, but not phagocytosed [17,72], and then cause bacteremia and even septemia.

One of the key questions to be answered is how *S. suis *crosses the blood-brain barrier to cause meningitis, which was observed in all WT-infected pigs. The findings of the reported study presented that suilysin-positive strain could show toxin to produce functional alteration and increase the permeability of BBB; and Suilysin-negative strain might stimulate the production of proinflammatory cytokines resulting in alteration of BBB permeability [76,77]. And they also indicated that this highly pathogenic strain could produce high level of toxins in vivo-Suilysin, MRP, hyl [74], and undoubtedly it would contribute to the penetration of deep tissue and BBB. In addition, the stimulated production of proinflammatory cytokines would result in the alteration of BBB permeability, and it would be more feasible for *S. suis *to break through BBB. From our understanding, WT strain could utilize the synergic effect of toxins and high level of cytokines to accelerate the penetration of deep tissue and BBB. These might be the reason why the strain could cause severe human diseases in Sichuan, 2005.

## Conclusions

Microarray technology has been used to analyse the globle porcine transcriptional response to infection with various pathogenic microorganisms recently. Study on the transcriptional response to the Gram-positive bacterium SS2 by using the Affymetrix GeneChip Porcine Genome Array has not been reported until now. Although great efforts have been made to understand the molecular basis of this infection, the response to SS2 infection is still largely unknown. Transcriptome analysis based on S. suis-infected spleens could improved the interference received by the cells analysis, and also supply the solid supplementary for analysis on alveolar macrophages. Highly pathogenic S. suis could persistently induce cytokines mainly by TLR2 pathway, and eventually the high level of cytokines and toxins secreted by phagocytosis-resistant bacteria could destroy deep tissues, and cause meningitis, septicaemia, pneumonia, endocarditis, and arthritis.

## Methods

### Bacterial strains

SS2 strain 05ZY (WT) which was isolated from the brain of a diseased piglet collected in Sichuan outbreak in China 2005 showed high virulence to pigs [4,78], and was applied to infect pigs. An isogenic *HP0197 *mutant (ΔHP0197) derived strain 05ZY showed no obvious virulence to pigs (unpublished data) was applied as a control.

### Animals infection and tissue collection

All the experimental protocols were approved by the Laboratory Animal Monitoring Committee of Hubei Province and performed accordingly. A total of 12 pigs of high-health status (ages 4-5 weeks) were assigned to three groups, within four in each. The pigs were determined to be SS2-free by antibody-based ELISA and nasal swabs-based bacteriologic test. One hour before inoculation, all pigs were given 2 ml of 1% acetic acid (pH 2.9) intranasally to enhance the sensitivity of the *S. suis *challenge. Two groups were inoculated intranasally with 1 ml of 2×10^6^CFU of WT strain or ΔHP0197 respectively, and the rest group inoculated with PBS was served as control. All pigs inoculated with WT showed typical symptoms at day 3 while pigs inoculated with ΔHP0197 or PBS showed no significant clinical signs. Blood samples from each group were detected for bacterial burden. Bacteria could be found in the blood of pigs in the WT group at day 3 post-inoculation while no bacterium was found from the blood of pigs inoculated with isogenic mutant strain or PBS at the same time point. All pigs were sacrificed at day 3, and their tissue samples were cultured to prove in vivo bacterial burden. Bacteria were found in the spleens of the WT group, and no bacterium was found in the other two groups. Spleen samples were aseptically collected and immediately frozen in liquid nitrogen for future RNA isolation. Total RNA was isolated from approximately 200 mg of each sample by using the TRIzol (Invitrogen) and RNeasy Midi kit (QIAGEN) based on the manufacturer's protocols. The integrity, quality, and quantity of RNA were assessed using the Agilent Bioanalyser 2100.

### Microarray hybridizations and data analysis

The RNA labelling and hybridization were conducted by a commercial Affymetrix array service (CapitalBio Corp. Beijing, China). An aliquot of 2 μg of total RNA was converted to double-stranded cDNA with the one-cycle cDNA Synthesis Kit (Affymetrix), and then biotin-tagged cRNA was produced with MessageAmp™ II aRNA Amplification Kit. The resulting bio-tagged cRNA was fragmented to strands of 35 to 200 bases in length according to Affymetrix's protocols and then it was hybridized to GeneChip Porcine Genome Array. Hybridization was performed at 45°C with rotation for 16 h (Affymetrix GeneChip Hybridization Oven 640). The GeneChip arrays were washed and then stained (streptavidin-phycoerythrin) on an Affymetrix Fluidics Station 450 followed by scanning on GeneChip Scanner 3000.

The hybridization data were analyzed using GeneChip Operating software (GCOS 1.4). A global scaling factor of 500 was used to normalize the different arrays. We identified the differentially expressed genes according to change p-value calculated by GCOS 1.4, and 2-fold change as an empirical criterion. Then all DE genes were performed for hierarchical cluster (Ver.3.0) and TreeView (Ver.1.1.1) analyses. Genes with significant similarities to transcripts in nr database based on BLASTX searches were selected for GO analysis with DAVID http://david.abcc.ncifcrf.gov/home.jsp. Annotation results were obtained by inputting the gene list of ENTREZ_GENE_ID as identifier. All microarray results from this study were deposited in NCBI's Gene Expression Omnibus (GEO) database, accession numbers are: Platform, GPL3533; Series, GSE23596; Samples, GSM578704, GSM578705, GSM578706, GSM578707, GSM578708, GSM578709, GSM578710, GSM578711, GSM578712.

### qPCR analysis

All tested RNAs from swine spleens were reversely transcribed to cDNA with the M-MLV Reverse Transcriptase (Promega). Each cDNA sample was used as a template for qPCR and the amplification mixture contained SYBR Green (TOYOBO, Japan), forward and reverse primers. Some primers were designed by the program Primer 5.0, the primer names, accession number, primer sequence and product size are shown in Table 3. The efficiency of the PCR reaction was 91-99% for all reactions (slope standard line between -3.3 and -3.6). The standard line consisted of five 10-fold dilutions of the samples. Analysis was performed using the ABI7500 Software (Applied Biosystems). PCRs were performed in ABI PRISM 7500 sequence detection system as follows: 1 cycle at 95°C for 10 min; 45 cycles at 95°C for 30 s, 60°C for 30 s and 72°C for 30 s. Melting curves were performed at the end of amplification for validating data quality by increasing the temperature from 65°C to 95°C, read every 0.2°C, hold 2 sec, then cooling at 25°C for 30 s. The PCR products were confirmed using agarose gel electrophoresis (1.5%). Amplification of the *gapdh *gene was used as internal control. All the tested genes are shown in Table 3. All reactions were performed in triplicate. For each run, to normalize the amount of sample cDNA added to each reaction, the *Ct *value of each test gene was subtracted by the *Ct *value of the endogenous control *gapdh *gene (delta *Ct *= *Ct *tested gene - *Ct gapdh*), and then for a comparison between the expression of the gene in treated samples and in control samples. The delta *Ct *values of the gene in treated samples were subtracted by the delta *Ct *value of the gene in control samples (delta-delta *Ct *= delta *Ct *treatment - delta *Ct *control). The fold changes were calculated by the formula of 2^-delta-delta *Ct *^described by Livak & Schmittgen [79]. Data were means ± SD of triplicate reactions for each gene transcript.

**Table 3:** Primers for qPCR

primer	Accession number	Sequence	Product size
*Plau*	NM_213945	Forward: CGAACTGTGGCTGTCT Reverse: AGCAGGTTTGCGATGTG	126 bp
*S100A9^a^*	BI402402	Forward: CCAGGATGTGGTTTATGGCTTTC Reverse: CGGACCAAATGTCGCAGA	186 bp
*S100A12^a^*	CB475695	Forward: GGCATTATGACACCCTTATC Reverse: GTCACCAGGACCACGAAT	169 bp
*Hsp70*	NM_213766	Forward: AGGCGGAGAAGTACAAAGCG Reverse: GATGGGGTTACACACCTGCTC	257 bp
*Timp1*	NM_213857	Forward: CGCCTCGTACCAGCGTTAT Reverse: GTGGAAGTATCCGCAGACGC	127 bp
*SOD2*	NM_214127	Forward: TCTGGACAAATCTGAGCCCT Reverse: GACGGATACAGCGGTCAACTT	119 bp
*Il6^b^*	AF493992	Forward: GACAAAGCCACCACCCCTAA Reverse: CTCGTTCTGTGACTGCAGCTTATC	69 bp
*Sele*	NM_214268	Forward: GGATTTGAACTCATCGGACCT Reverse: CATTCTGAGGATGGCCGAC	115 bp
*Il1b^b^*	NM_214055	Forward: GGCCGCCAAGATATAACTGA Reverse: GGACCTCTGGGTATGGCTTTC	70 bp
*Nramp1*	U55068	Forward: CGTGGTGACAGGCAAGGACT Reverse: TAGCCGTGCCGATGACTTC	131 bp
*Hsp90*	CF180819	Forward: CCCAGTTGATGTCGTTG Reverse: CCGTCAGGCTTTCGTAT	117 bp
*Il8 ^b^*	NM_213867	Forward: TTCGATGCCAGTGCATAAATA Reverse: CTGTACAACCTTCTGCACCCA	176 bp
*CCL19*	BX672579	Forward: GCTAAGCCTCTGGACT Reverse: AATGAGCAGGTAGCGA	121 bp
*Haptocorrin*	CB472702	Forward: ATTCTCAGGGAGTATTCCGTCT Reverse: CTTTGGGGACAAGTAGCAGTT	105 bp
*Alox15*	NM_213931	Forward: ACCGAGGGTTTCCTGTCT Reverse: AGGTGGTTGGAGGAGTGC	100 bp
*TLR2^b^*	NM_213761	Forward:TCACTTGTCTAACTTATCATCCTCTTG Reverse: TCAGCGAAGGTGTCATTATTGC	162 bp
*GAPDH ^b^*	AF017079	Forward: TGCCAACGTGTCGGTTGT Reverse: TGTCATCATATTTGGCAGGTTTCT	62 bp

*a*: primers from reference [43];

*b*: primers from reference [41].

## Abbreviations

SS2: *Streptococcus suis *serotype 2; DE: differentially expressed; FC: fold change; GO: Gene Ontology; FDR: false discovery rate; qPCR: quantitative real-time PCR; TLR: Toll-like receptors; PRRs: pattern-recognition receptors;

## Authors' contributions

RL and BC carried out all works and drafted the manuscript. AZ made substantial contributions to bioinformatics and statistical analysis. LT and YW participated in the animal challenge experiment. HC participated in the experiment design and coordination. MJ helped to revise and finalize the manuscript. All authors read and approved the final manuscript.

## Supplementary Material

Additional file 1 **Spleen transcriptome analysis following *S. suis *infection using the Affymetrix Porcine Genechip**. Data of each probe is from the three piglets of the control group (NC-1P, NC-2P, NC-3P), the WT group (WT-1P, WT-2P, WT-3P) and the △HP0197 group (M97-1P, M97-2P, M97-3P). "P", present; "A", absent; "M", marginal; select while Count (P) ≥ 2 in two groups. Totally, 15,757, 14,992 and 15,487 probesets were detected expression in the control group, the WT group and △HP0197 group respectively. Click here for file

Additional file 2 **transcripts expressed in porcine spleen following *S. suis *(WT) infection**. "FC", Fold change, gene expression level following WT infection compared to the control. "≥2" represents up regulation, " < 1" represents down regulation. "q-value", significance level of differential expression for a particular gene. "Gene description", top informative BLASTX hit. Click here for file

Additional file 3 **Differentially expressed transcripts in porcine spleen following *S. suis *(WT) infection (q < 10%, FC≥2)**. 120 transcripts (row 5-124) were significantly up-regulated, and 132 (row 125-256) were significantly down-regulated, "FC", Fold change, gene expression level following WT infection compared to the control. Click here for file
